# Transcriptome sequencing of *Coccinella septempunctata* adults (Coleoptera: Coccinellidae) feeding on artificial diet and *Aphis craccivora*

**DOI:** 10.1371/journal.pone.0236249

**Published:** 2020-08-17

**Authors:** Ying Cheng, Junrui Zhi, Fengliang Li, Hua Wang, Yuhang Zhou, Jianxue Jin

**Affiliations:** 1 Institute of Entomology, Guizhou Provincial Key Laboratory for Agricultural Pest Management of the Mountainous Region, Guizhou University, Guiyang, China; 2 Institute of Plant Protection, Guizhou Academy of Agricultural Sciences, Guiyang, China; 3 Guizhou Institute of Forest Inventory and Planning, Guiyang, China; Universite de Lausanne Faculte de biologie et medecine, SWITZERLAND

## Abstract

**Background:**

The insect predator *Coccinella septempunctata* can effectively control many types of pests, such as aphids, whiteflies, and small lepidopteran larvae. We previously found that *C*. *septempunctata* fed an artificial diet showed diminished biological properties(e.g. fecundity, egg hatching rate, survival rate, etc.) compared with those fed natural prey (*Aphis craccivora*), likely due to different nutritional characteristics of the diet. In this study, we used transcriptome sequencing analysis to identify nutrition- and metabolism-related genes of *C*. *septempunctata* that were differentially expressed depending on diet.

**Methodology/Principal findings:**

The Illumina HiSeq2000 was used to sequence 691,942,058 total clean reads from artificial diet-fed and *A*. *craccivora*-fed *C*. *septempunctata* libraries, and the clean reads were assembled using Trinity *de novo* software (Tabel 2). Comparison of transcriptome sequences revealed that expression of 38,315 genes was affected by the artificial diet, and 1,182 of these genes showed a significant change in expression levels (FDR ≤ 0.05,|log2FC|≥1, “FC” stands for “fold change”). These differentially expressed genes (DEGs) were likely associated with the decreased egg laying capacity, hatching rate, longevity, and increased sex ratio (♀:♂) of adult *C*. *septempunctata* observed in the group fed the artificial diet. Furthermore, in the most DEGs metabolic pathways for *C*. *septempunctata* feeding on the artificial diet accumulated amino acid metabolic pathways, lipid metabolic pathways, and starch and glucose metabolism were down-regulated.

**Conclusions/Significance:**

We found some differentially expressed genes and metabolic pathways are related to nutrition, from which a more informative feedback for diet formulation was obtained and the artificial diet could be more efficiently optimized.

## Introduction

The lady beetle, *Coccinella septempunctata* L. (Coleoptera: Coccinellidae), is a natural enemy of insect pests. It has received increasing attention as a bio-control agent due to its strong predatory ability against many important types of pests, including aphids, whiteflies, spider mites, leafhoppers, and psyllids [1–8]. Because climate, farming operations, and abuse of pesticides make it difficult to maintain sufficient numbers of *C*. *septempunctata* in the field to control pests effectively, it is necessary to rear them artificially. Currently, *C*. *septempunctata* is reared mainly using aphids as food, but this method is costly and time-consuming because it requires mass rearing of both aphids and *C*. *septempunctata* [9–10]. Therefore, the availability of artificial diets is crucial for successful mass rearing of *C*. *septempunctata* at a commercial level.

Since the 1950s [11], researchers have been trying to develop an effective artificial diet for rearing ladybug, it was still difficult that can reproduce these insects at a large scale. Understanding the insect diet is difficult because the physiological and biochemical processes that regulate insect populations are poorly known, and physiological or biochemical markers that can be used to evaluate the suitability of a particular type of nutrition are lacking. Nutrigenomics examines how nutrition affects gene expression patterns, and it offers a means to measure an insect’s response to changes in the food stream and can provide information about diet limitations [12]. Such studies revealed that *Drosophila melanogaster* and *Caenorhabditis elegans* expressed similar components in the insulin signaling pathway [13,14]. Zinke et al. demonstrated that in *Drosophila* larvae, different nutritional conditions have distinct effects on gene expression patterns, and lipase-3 and enolpyruvate carboxylic kinase are up-regulated during starvation [15]. In another study, Zinke et al. divided the differentially expressed genes (DEGs) that control nutrition in the *Drosophila* nymph into groups of different physiological metabolic pathways that regulate glucose metabolism, lipid metabolism, and cell growth [16]. They found that some genes were upregulated or downregulated during the one hour of nutrient deprivation. Using suppressive subtractive hybridization, Yocum et al. discovered two artificial diets upregulated and two prey upregulated transcript fragments in the predatory pentatomid *Perillus bioculatus*, and a BLASTx search found similarities for two diet upregulated clones (i.e., the tyrosine-3-monooxygenase gene and the chitin binding protein gene, Gasp) [12]. Alaux et al. compared differences in the transcriptome sequence of bees fed on pollen and sugar versus those fed a diet lacking sugar and reported that pollen activates the metabolic pathways of sensitive nutrition [17]. Coudron et al. found that the level of trace elements in *Podisus maculiventris* is essentially influenced by food sources [18]. Zou et al. reported that many metabolic pathways related to nutrition were upregulated in diet-fed *Arma chinensis*, which in some cases indicated excess quantities of specific nutrients in the diet [19]. They showed that changes in gene expression caused by dietary changes were correlated with physiological differences observed in diet-fed *A*. *chinensis* and Chinese oak silk moth pupae fed *A*. *chinensis*. Li et al. showed that the citrus mealybug *Planococcus citri* were less suitable to *Cryptolaemus montrouzieri* compared to the aphid *Megoura japonica*, and the observed up-regulation of genes related to biochemical transport and metabolism and detoxification were probably a result of adaptation to the changes in nutritional and non-nutritional (toxic) components of the prey [20].

These studies showed that it is feasible to use nutritional genomics to analyze artificial diets of insects and to use the results to formulate better artificial diets. To date, however, nutritional genomics analysis has not been used to study the artificial diet of *C*. *septempunctata*. The goals of this study were to use the Illumina HiSeq2000 transcriptome sequencing technology to analyze the differences in gene expression and nutrient metabolic pathways between *C*. *septempunctata* fed an artificial diet and those fed on aphids and to provide a reference for improving the artificial diet of *C*. *septempunctata*.

## Materials and methods

### Insects

The *C*. *septempunctata* colonies used in this study were originally obtained from the experimental fields of the Guizhou Provincial Academy of Agricultural Sciences, Guiyang, China. The insects used in this experiment were reared at 25 ± 1°C, relative humidity of 70 ± 5%, and 14 hours of light and 10 hours of darkness a day. The prey, *A*. *craccivora*, were maintained on horsebean seedlings in the laboratory. The artificial diet was consisted according to Table 1. Adults used in this study were 30 day old, and 30 female(15 from aphid-fed and 15 from artificial diet-fed) and 30 male(15 from aphid-fed and 15 from artificial diet-fed) adults were collected for RNA extraction. Total RNA was extracted from freshly sacrificed insects.

**Table 1:** Composition of the artificial diet used to rear *C*. *septempunctata*.

Ingredient	Amount	Ingredient	Amount
Raw pork liver	105.00 g	Vitamin powder	1.50 g
Milk powder	15.00 g	65% juvenile hormone Ⅲ	4.50 μl
Sucrose	30.00 g	Protein powder	3.00 g
Olive oil	3.00 ml	Vitamin E	0.75 ml
Yolk	15.00 g	Honey	7.50 g
Corn oil	2.00 ml	Pumpkin	10.00 g
Yeast powder	5.00 g	Sorbic acid	1.00 g
Cholesterol	0.50 g	Agar	6.24 g
Casein	5.00 g	Distilled water	374.49 ml
Casein hydrolyzate	4.80 g	—	—

### cDNA library construction, and sequencing

Total RNA was extracted using TRIzol^®^ Reagent (Invitrogen, Carlsbad, CA, USA) following the manufacturer’s instructions. Gene expression information was obtained from RNA samples from male and female adults of the F8 inbreeding generation fed on artificial diet or aphids. RNA sequencing was performed by the Majorbio Biotechnology Corporation (Shanghai, China) using a 2100 Bioanalyzer (Agilent Technologies, Santa Clara, CA, USA) and following the Illumina manufacturer’s instructions. The poly(A)+RNA was purified using oligo(dT) magnetic beads and was fragmented into short sequences in the presence of divalent cations. The cleaved poly(A)+RNA was reverse transcribed to cDNA. After end repair and ligation of adaptors, the products were amplified by polymerase chain reaction (PCR) to create a cDNA library, which was sequenced on an Illumina HiSeq2000 DNA sequencer (San Diego, CA, USA).

### Sequence data processing and *de novo* assembly

**Fig 1:**
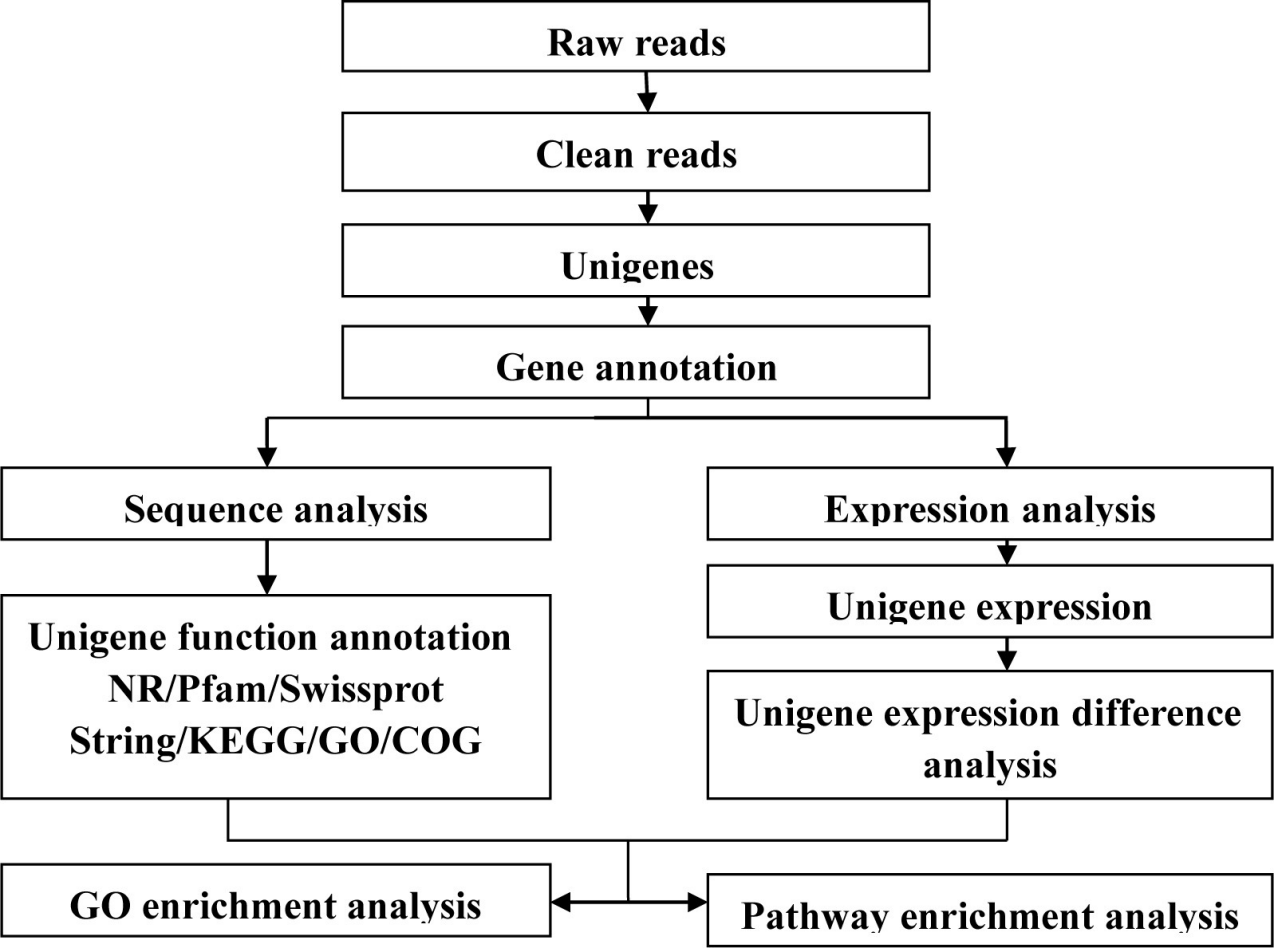
Pipeline of bioinformatics analysis.

Image deconvolution and quality value calculations were performed using Illumina HCS 1.1 software. Transcriptome raw sequences were subjected to a series of assembly and annotation programs (Fig 1). *De novo* assembling of short reads originating from Illumina sequencing was performed using Trinity [21]. Before assembly, raw reads were trimmed by stripping the adaptor sequences and ambiguous nucleotides using SeqPrep (https://github.com/jstjohn/SeqPrep) and Sickle

(https://github.com/najoshi/sickle).

### Functional unigene annotation and classification

The resulting transcripts from Trinity assembly were used as queries for open reading frame (ORF) prediction. The default sequence contains a untranslated regions (UTR) and the Markov model was used to predict the best ORF region in this sequence, then the pfam database was used to correct the prediction results, and the protein sequences BLAST to the database were retained. Sequence homology searches of the protein sequences with predicted ORFs were performed using the HMMER3 program against sequences in the Pfam database (http://pfam.sanger.ac.uk/), while the contigs without predicted ORFs were used as queries for BLASTX searches [22]. The cutoff Expect value (E-value) was set at 1𝑒^–5^, and only the top hit result against known sequences was assigned as the annotation. Gene ontology (GO) analysis for biological process, molecular process, and cellular component was conducted using the Blast2GO program [23]. All annotated contigs were categorized with regard to biological process, cellular component, and molecular function. They were used to determine the GO terms and clusters of orthologous groups (COG) terms and for further Kyoto encyclopedia of genes and genomes (KEGG) pathway analysis with Blast.

### DEGs and enrichment analysis

The fragments per kb per million fragments (FPKM) [24] was used to calculate unigene expression [25]. The false discovery rate (FDR) method was utilized to determine the threshold p-value in multiple tests as follows: If the FDR was < 0.05 and FPKM values showed at least a two-fold difference between the two samples, this unigene was considered to be a significant DEG. The DEGs were used for GO and KO enrichment analyses. P-values were calculated according to the hypergeometric test:

$$P=1‐\sum_{i=0}^{m-1}\frac{\left(\begin{array}{c} M\\ i \end{array}\right)\left(\begin{array}{c} N-M\\ n-i \end{array}\right)}{\left(\begin{array}{l} N\\ n \end{array}\right)}$$

where N represents the number of genes with GO/KO annotation, n represents the number of differentially expressed genes in N, M represents the number of genes in each GO/KO term, and m represents the number of differentially expressed genes in each GO/KO term. GO and KEGG pathway enrichment analyses were conducted by hypergeometric distribution testing using the software GOatools (https://github.com/tanghaibao/GOatools) and KOBAS (http://kobas.cbi.pku.edu.cn/home.do), respectively. Bonferroni correction was used to adjust p-values. Significantly enriched functional clusters were identified when the corrected p-value was < 0.05.

### Identification of Simple Sequence Repeats (SSRs) and Single Nucleotide Polymorphisms (SNPs)

SSRs were identified using the microsatellite identification tool (MISA) http://pgrc.ipk-gatersleben.de/misa/misa.html). The candidate SNPs were identified in the assembled sequences of *C*. *septempunctata* using Samtools [26] and VarScan [27] software.

### Quantitative real-time PCR (qRT-PCR) validation

qRT-PCR was performed on 14 randomly selected DEGs with two biological replicates and three technical replications. Total RNA was extracted as described for the DEG library preparation and sequencing. The 18S rRNA was used as the internal reference gene. The selected reagents used were PrimeScript™ RT reagent kit with DNA Eraser (Takara: RR047A) and Bestar^®^ SybrGreen qPCR MasterMix (Germany: DBI-2043). The qRT-PCR system (20 μl) consisted of the following: 10 μl of Bestar® SybrGreen qPCR mastermix, 2 μl of template cDNA, 0.25 μl (0.25 μM) of upstream and downstream primers, 0.04 μl of 50× ROX, and 7.46 μl of RNase-free water. The cycling parameters were 95°C for 2 min followed by 45 cycles at 95°C for 10 s, 60°C for 34 s, and 72°C for 30 s, ending with a melting curve analysis (60–95°C) to check for nonspecific product amplification. Relative gene expression was analyzed using the 2^–ΔΔCT^ method [28].

## Results and discussion

### Illumina sequencing and *de novo* assembly

Using the sequencing data obtained through the Illumina HiSeq 2000 platform, we performed routine transcriptome analysis on the following groups of adult *C*. *septempunctata*: artificial diet-fed females (ADF), artificial diet-fed males (ADM), aphid-fed females (CKF), and aphid-fed males (CKM). With three biological replicates of the four sample groups, the 12 libraries yielded 701,209,664 raw reads, comprising 184,211,172, 180,7128,848, 168,513,480, and 167,772,164 reads for the ADF, ADM, CKF, and CKM groups, respectively (Table 2). After adaptor trimming and quality filtering, 691,942,058 clean reads were filtered and 134,831 contigs were assembled, with a mean length of 795 bp and an N50 of 1,648 bp (i.e., 50% of assembled bases were incorporated into contigs of 1,648 bp for insects) (Table 2). The contigs were assembled into 100,733 unigenes with an average length of 689 bp (S1 File). The lengths of the assembled unigenes were primarily in the range of 1 to 200 bp (approximately 64.71% of the total unigenes were in this range), with unigenes falling between 401 and 600 bp (12.09%).

**Table 2:** Summary statistics from Illumina sequencing of the *C*. *septempunctata* L. transcriptome.

Sequencing	
Number of raw reads	701, 209, 664
Number of clean reads	691, 942, 058
Total number of contigs	134, 831
Total length of contigs (bp)	107, 166, 310
Contig N50	1, 648
Mean length of contigs (bp)	795
Total number of unigenes	100, 733
Total length of unigenes (bp)	69, 384, 106
Unigenes of N50	1, 402
Mean length of unigene (bp) Annotations	689
Unigene annotations against Nr	20, 092
Unigene annotations against Swissport	15, 594
Unigene annotations against String	7, 805
Unigene annotations against KEGG	14,414
Unigene annotations against COG	4,652
Unigene annotations against GO	9, 304

### Annotation of unigenes

For functional annotation, distinct gene sequences were searched using BLASTX against Nr, Swissport, String, KEGG, COG, and GO (E-value < le^–5^): 20,092 of 100,733 unigenes were annotated against Nr (approximately19.95% of all of the unigenes) (Table 2), In total 9,304 unigenes of *C*. *septempunctata* were assigned to GO terms based on BLAST matches with previously known sequences. These unigenes were associated with biological processes (25 sub-categories, 25,784 sequences), cellular components (20 sub-categories, 15,114 sequences), and molecular functions (18 sub-categories, 12,046 sequences) (S2 File). Six major sub-types were metabolic processes (5,926, 22.98%), cellular processes (5,440, 21.10%), cell (3,257, 21.55%), cell part (3,257, 21.55%), binding (4851, 40.27%), and catalytic activity (4,791, 39.77%). Among the molecular functions, channel regulator activity (1, 0.01%), metallochaperone activity (1, 0.01%), and morphogen activity (1, 0.01%) were the least abundant categories.

We searched the annotated sequences for genes involved in COG classifications. From 20,092 Nr hits, 4,652 sequences had a COG classification (S3 File). Among the 25 COG categories, the cluster for general function prediction only represented the largest group (669, 14.38%), followed by translation, ribosomal structure, and biogenesis (589, 12.66%), posttranslational modification, protein turnover, chaperones (460, 9.89%), and signal transduction mechanisms (386, 8.30%). Nuclear structure (3, 0.06%) and cell motility (11, 0.24%) represented the smallest groups. Extracellular structures did not match any sequences.

To identify the biological metabolic pathways in *C*. *septempunctata*, we mapped the 134,831 contig sequences to the reference canonical pathways in the KEGG database. A total of 19,142 sequences were annotated in KEGG and located to 379 known KEGG pathways (S4 File). The pathways most represented by the unique sequences were metabolic pathways (3,149, 16.45%), biosynthesis of secondary metabolites (1,285, 6.71%), biosynthesis of antibiotics (964, 5.04%), and microbial metabolism in diverse environments (932, 4.87%). The metabolic pathways included amino acid metabolism(986), starch and sucrose metabolism(189), fatty acid metabolism(162) and linoleic acid metabolism(10). The biosynthesis included amino acids biosynthesis(436), fatty acids biosynthesis(58), starch and sucrose biosynthesis(45), insect hormone biosynthesis(33), fat digestion and absorption(30) and vitamin digestion and absorption(16). These annotations provide a valuable resource for investigating specific processes, functions, and pathways during nutrigenomics research of *C*. *septempunctata*.

### DEG statistics

**Fig 2:**
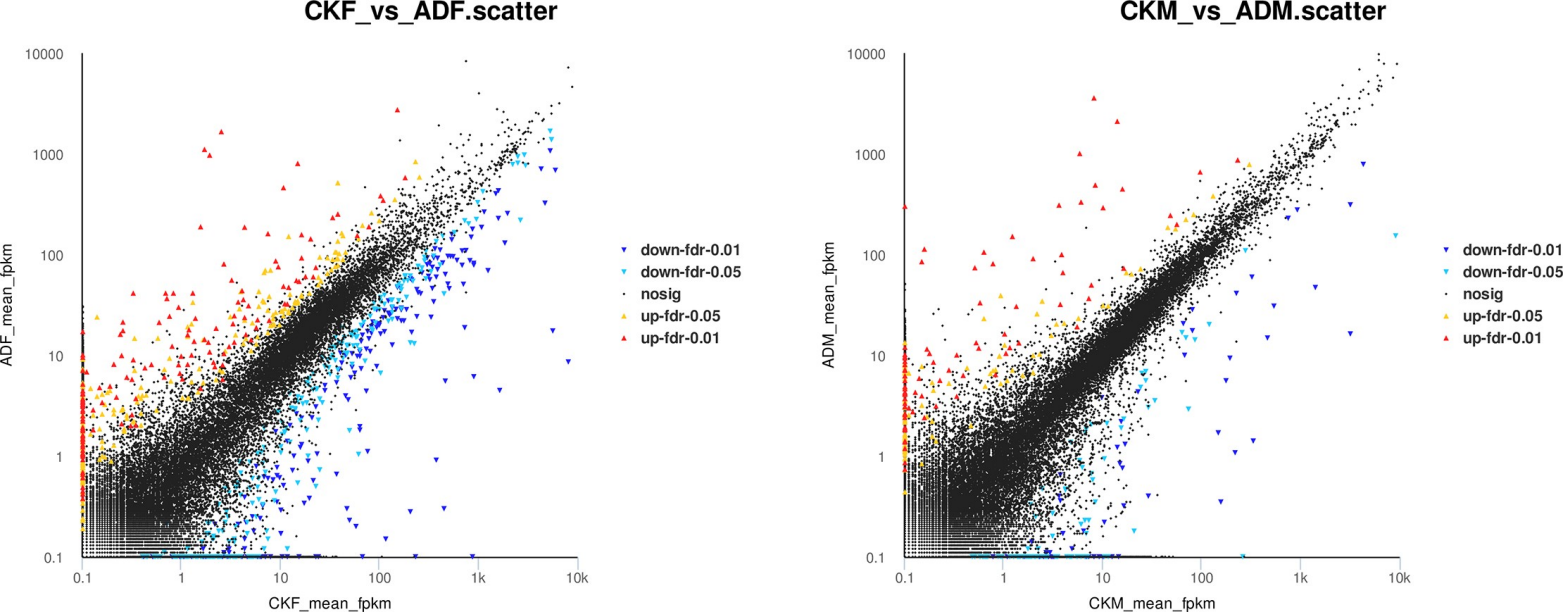
Expression level of the differentially expressed genes in artificial diet-fed vs. prey-fed treatments for (A) female and (B) male *C*. *septempunctata*.

The expression levels of 38,315 genes were affected by the artificial diet, and 1,182 genes showed significantly different expression levels (FDR ≤ 0.05 and |log2FC| ≥ 1) between the two food treatments. Among those, 342 were upregulated and 489 were downregulated in the ADF group compared to the CKF group (Fig 2A, S5 File), and 182 were upregulated and 169 were downregulated in the ADM group compared to the CKM group (Fig 2B, S5 File). Of the 30 most differentially upregulated female ladybug genes, 11 had defined functions (i.e., three major royal jelly proteins, one calcineurin-like phosphoesterase, one acyltransferase family member, one reverse transcriptase [RNA-dependent DNA polymerase], one PIF1-like helicase, one sugar transporter, one helitron helicase-like domain at N-terminus, one partial alpha/beta-hydrolase lipase region, and one lipase). Of the 30 most differentially downregulated female ladybug genes, 11 had defined functions (i.e., one gag-polypeptide of LTR copia-type, one RNA dependent RNA polymerase, one PBP/GOBP family member, one transposase protein, one leucine-rich repeat, one carboxylesterase family, one lytic polysaccharide mono-oxygenase, one kazal-type serine protease inhibitor domain, one reverse transcriptase [RNA-dependent DNA polymerase], one polypreny synthetase, and one immunoglobulin domain) (S6 File). Of the 10 most differentially upregulated male ladybug genes, 3 had defined functions (i.e., one membrane-bound O-acyltransferase family, one cytochrome P450, and one cytosolic fatty-acid binding protein family member). Of the 10 most differentially downregulated male ladybug genes, five had defined functions (i.e., two transposase proteins, one gag-polypeptide of LTR copia-type, one apolipophorin-III precursor, and one PBP/GOBP family member) (S6 File). These top 80 most differentially up- and downregulated genes demonstrated that the nutritional differences between aphids and the artificial diet caused changes in a broad range of genes, yet both supported complete development of *C*. *septempunctata*.

#### GO assignments of DEGs

To understand the functions of the DEGs, 657 DEGs of *C*. *septempunctata* were assigned to GO terms based on BLAST matches with sequences of known function. For the ADF/CKF and ADM/CKM groups, 495 GO terms (including 239 biological processes, 149 molecular functions, and 107 cellular components) (S7 and S8 Files) and 162 GO terms (including 72 biological processes, 53 molecular functions, 37 cellular components) (S9 and S10 Files), respectively, classified all of the DEGs.

### KEGG of DEGs

To identify the biological pathways associated with genes differentially expressed between the food treatments, we mapped the 1,182 DEGs (FDR ≤ 0.05 and |log2FC| ≥ 1) and annotated sequences to the reference normative pathways in KEGG. We assigned 510 sequences to 267 KEGG pathways. The pathways most represented by the DEGs were metabolic pathways, microRNAs in cancer, ubiquitin-mediated proteolysis, RNA transport, the NF-kappa B signaling pathway, biosynthesis of secondary metabolites, purine metabolism, biosynthesis of antibiotics, and fat digestion and absorption in ADF/CKF (S11 File) and ADM/CKM (S12 File). These annotations provide a valuable resource for investigating specific processes, functions, and pathways during nutrigenomics research of *C*. *septempunctata*.

### DEGs related to different biological characteristics

Several biological characteristics differed between aphid-fed and diet-fed *C*. *septempunctata*, such as reduced fecundity, lower egg viability, increased sex ratio (♀:♂), and shorter lifespan. We found several DEGs (FDR ≤ 0.05 and |log2FC| ≥ 1) that were related to these biological characteristics (S13 File). For reduced fecundity, five haemolymph juvenile hormone binding proteins(*protein takeout-like*, *conserved hypothetical protein*, *circadian clock-controlled protein*, *hypothetical protein TcasGA2_TC001181*, *protein takeout-like*) and two male sterility proteins(*AGAP011736-PA-like protein、putative fatty acyl-CoA reductase CG5065*) were markedly downregulated in diet-fed female insects. It may be that the amount of juvenile hormone in diet was insufficient. Juvenile hormones affect development of the female reproductive system and vitellogenesis in a variety of insects [29–31], and expression of vitellogenin genes in fat bodies of adult female *C*. *septempunctata* is regulated by juvenile hormone [32]. In addition, vitellogenesis in insects is regulated by endocrine hormones, and the nutrition factor also plays an important role in most female insects [33]. The absence of one or more nutrients can affect the transcriptional synthesis of vitellogenin. We found that three vitellogenin genes were markedly upregulated in diet-fed adult female *C*. *septempunctata* compared to aphid-fed females, which indicates that the nutrition in the artificial diet is suitable for vitellogenesis.

Among the genes that affect insect longevity, the gene copper/zinc superoxide dismutase was downregulated in diet-fed *C*. *septempunctata* [34], which shortened their lifespan relative to aphid-fed individuals. Cytochrome P450 genes are involved in synthesis of steroid hormones (ecdysone) in organisms, the ecdysis of insects is regulated by ecdysone. The six cytochrome P450 genes (*cytochrome P450 345D2*, *cytochrome P450 CYP4BN1*, *cytochrome P450 307A1*, *cytochrome P450 CYP6BQ21*, *cytochrome P450 315a1*, *cytochrome P450*) were upregulated in diet-fed *C*. *septempunctata*, these upregulated genes may have contributed to the observed prolonged larvae development time in this treatment group. In addition, three major royal jelly proteins (MRJPs) were significantly upregulated in the diet-fed group. These proteins are related to the YELLOW family [35], whose members promote individual sexual maturity and development of different sex [36]. At the same time, the MRJPs as the main nutritional components of royal jelly decided the classification of bees, MRJPs also may promote maturity of specific sex [37–39]. Therefore, the increase of sex ratio of diet-fed *C*. *septempunctata* may be associated with adding honey in diet. In addition, we found that expressions of four PBP/GOBP family genes (two *general odorant-binding protein 19d-like* and two *Tribolium odorant binding protein*), PBP is a pheromone binding protein, and GOBP is a general odorant-binding protein; and three insect pheromone-binding family genes *(putative chemosensory binding protein*, *chemosensory protein 12 precursor*, and *putative chemosensory binding protein)* were downregulated (S13 File). Thus, the foraging behavior, alertness, and mating ability of diet-fed *C*. *septemtmnctata* may be reduced.

### KEGG of DEGs related to artificial diets

Most DEGs enriched in the eight pathways related to amino acid metabolism (glycine, serine, and threonine metabolism; alanine and leucine biosynthesis; cysteine and methionine metabolism; lysine biosynthesis; tryptophan metabolism; glutathione metabolism; lysine degradation; and biosynthesis of amino acids) were downregulated in diet-fed insects (S14 File). Among the amino acid nutrition determination results, only the aspartic acid, tyrosine, and methionine content of the artificial diet were lower than the aphids. Although a large number of protein amino acids are added in the artificial diet, they exist mainly in the form of compounds. This result indicated that the amino acid digestion to absorption utilization ratio was poor for *C*. *septempunctata* fed the artificial diet, and thus the amino acid composition in the artificial diet needs further improvement.

In the seven pathways related to fat metabolism, most DEGs involved in the adipocytokine signaling pathway, pyruvate metabolism, fatty acid biosynthesis, glycerolipid metabolism, fatty acid metabolism, and biosynthesis of unsaturated fatty acids, were downregulated in diet-fed *C*. *septempunctata* (S14 File). This indicated that the artificial diet may contain insufficient lipids, suggesting that increases in the olive oil, corn oil, and pig liver content are needed.

Most DEGs in the two pathways related to starch and sugar metabolism, and carbohydrate digestion and absorption were downregulated in diet-fed insects (S10 File). This indicated that the artificial diet may contain insufficient sugar.

Six metabolic pathways were associated with vitamins (ascorbate and aldolate metabolism; vitamin digestion and absorption; folate biosynthesis; retinol metabolism; one carbon pool by folate; and porphyrin and chlorophyll metabolism) (S14 File). Most DEGs involved in folate biosynthesis and vitamin digestion and absorption were downregulated in diet-fed insects. This indicated that the artificial diet may contain insufficient vitamins or that the vitamin powder added to the diet was not suitable for the digestion and absorption by *C*. *septempunctata*.

### qRT-PCR validation

**Fig 3:**
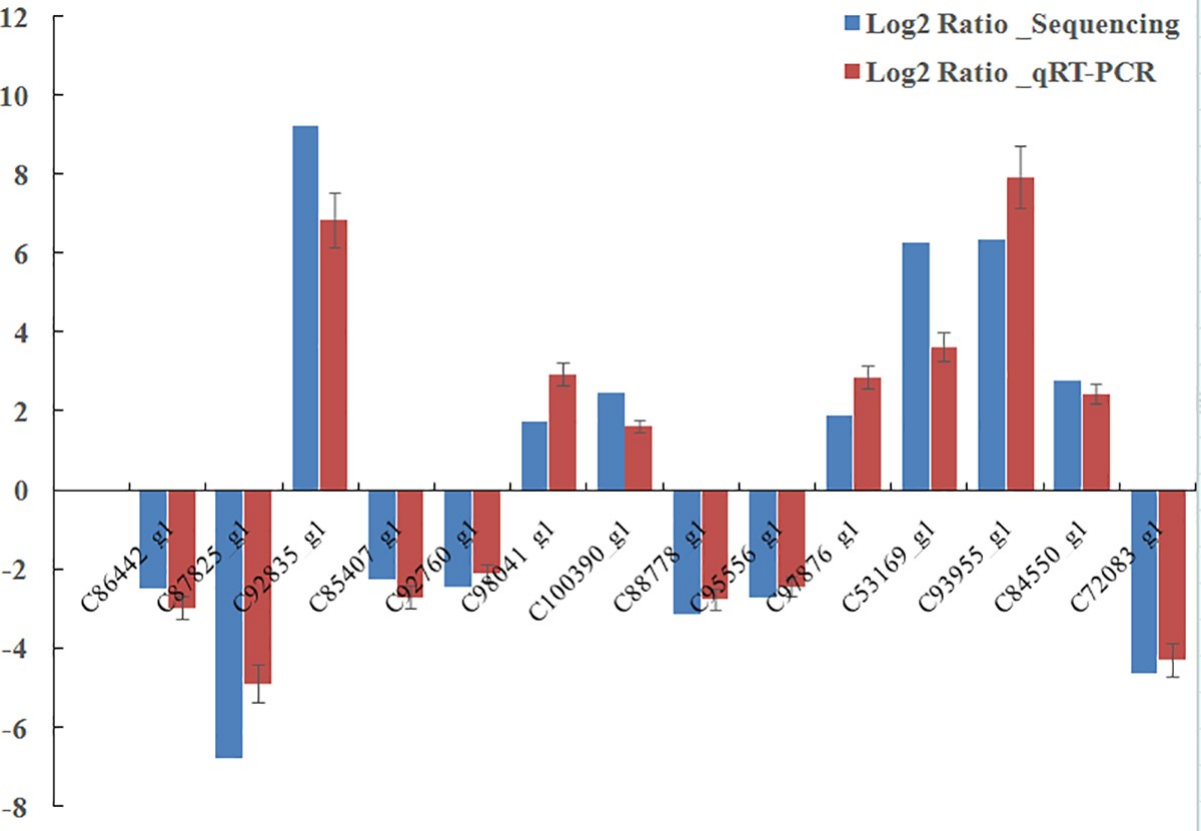
Verification of differentially expressed genes by qRT-PCR.

To validate the DEGs identified by the transcriptome analysis, we compared expression profiles of the ADF/ADM and CKF/CKM using qRT-PCR. We selected 14 genes randomly, and all of them demonstrated a concordant direction of change for both DEG and qRT-PCR analyses (Table 3). Both methods indicated that six genes (polysaccharide mono-oxygenase, gag-polypeptide of LTR copia-type, ribosomal protein, glycogen binding subunit 76A, glycosyl hydrolases family 18,16, and serine protease P61) were downregulated in the ADF compared to the CKF group. Three genes (major royal jelly protein, lipoprotein amino terminal region, and vitellogenin receptor) were upregulated in the ADF group compared to the CKF group. The putative chemosensory binding protein gene was downregulated and four genes (alpha-amylase catalytic domain a, cytochrome P450, membrane-bound O-acyltransferase family member, and cytosolic fatty-acid binding protein family member) were upregulated in the ADM compared to the CKM group. Among the 14 genes, there was no significant difference between the qRT-PCR and transcriptome sequencing results in terms of upregulated or downregulated values(Fig 3). Thus, the verification results of qRT-PCR confirmed the reliability of transcriptome sequencing.

**Table 3:** Verification of differentially expressed genes by qRT-PCR.

Gene name	Gene ID	Log2 Ratio _Sequencing	Log2 Ratio _qRT-PCR
Female			
*Polysaccharide mono-oxygenase*	C86442_g1	-2.48	-2.97
*gag-polypeptide of LTR copia-type*	C87825_g1	-6.79	-4.89
*Major royal jelly protein*	C92835_g1	9.25	6.85
*Ribosomal protein*	C85407_g1	-2.25	-2.70
*glycogen-binding subunit 76A*	C92760_g1	-2.42	-2.08
*Lipoprotein amino terminal region*	C98041_g1	1.75	2.93
*vitellogenin receptor*	C100390_g1	2.49	1.62
*Glycosyl hydrolases family 18*,*16*	C88778_g1	-3.13	-2.75
*serine protease P61*	C95556_g1	-2.71	-2.43
Male			
*Alpha-amylase catalytic domainɑ*	C97876_g1	1.89	2.87
*Cytochrome P450*	C53169_g1	6.27	3.62
*membrane-bound O-acyltransferase family*	C93955_g1	6.35	7.94
*cytosolic fatty-acid binding protein family*	C84550_g1	2.77	2.45
*putative chemosensory binding protein*	C72083_g1	-4.61	-4.28

## Conclusions

In this study, transcriptome sequencing of *C*. *septempunctata* feeding on an artificial diet versus an aphid diet revealed that the expression of 38,315 genes was affected by the artificial diet, and 1,182 of these genes showed a significant difference in expression level. These DEGs were likely associated with the decreased egg laying capacity, hatching rate, longevity, and increased sex ratio (♀:♂) of adult *C*. *septempunctata* feeding on the artificial diet. Genes for five hemolymph juvenile hormone binding proteins and two male sterility proteins were downregulated, and genes for six cytochrome P450 and three MRJPs were upregulated in female adult *C*. *septempunctata* fed on the artificial diet. Furthermore, in the most DEGs metabolic pathways for *C*. *septempunctata* feeding on the artificial diet accumulated amino acid metabolic pathways, lipid metabolic pathways, and starch and glucose metabolism were downregulated. In conclusion, we identified some differentially expressed nutrient-controlled genes and metabolic pathways that are related to nutrition in *C*. *septempunctata*, and these results can be used to formulate a better artificial diet for rearing this pest-control model.

## Supporting information

S1 File (XLS)

S2 File (XLS)

S3 File (XLS)

S4 File (XLS)

S5 File (PDF)

S6 File (DOC)

S7 File (XLS)

S8 File (XLS)

S9 File (XLS)

S10 File (XLS)

S11 File (XLS)

S12 File (XLS)

S13 File (DOC)

S14 File (DOC)
