# Supplementary material for: Transcriptome sequencing of Coccinella septempunctata adults (Coleoptera: Coccinellidae) feeding on artificial diet and Aphis craccivora
Source: PLoS One. 2020 Aug 17;15(8):e0236249. doi: 10.1371/journal.pone.0236249 (PMC7430724; doi:10.1371/journal.pone.0236249)
Supplement: S5 File — (PDF) [file pone.0236249.s005.pdf]

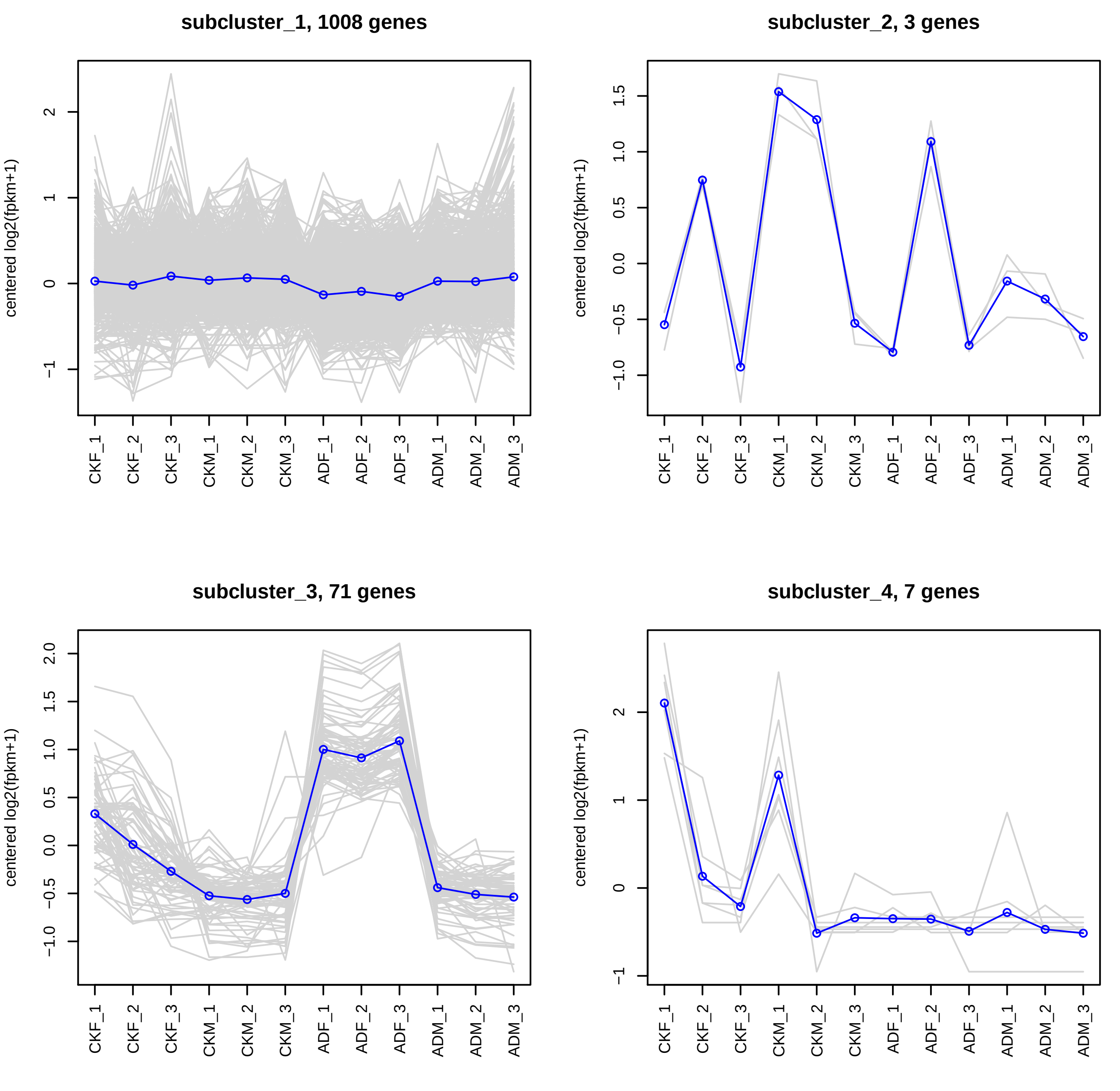
subcluster_1, 1008 genes
subcluster_2, 3 genes
subcluster_3, 71 genes
subcluster_4, 7 genes
centered log2(fpkm+1)
CKF_1
CKF_2
CKF_3
CKM_1
CKM_2
CKM_3
ADF_1
ADF_2
ADF_3
ADM_1
ADM_2
ADM_3

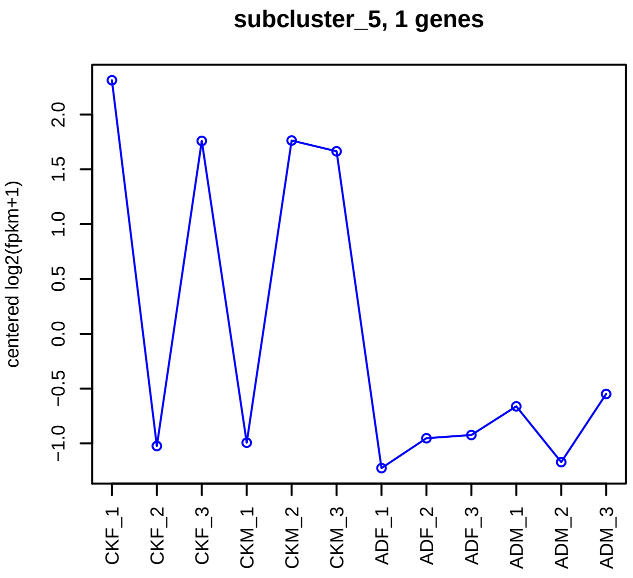
subcluster_5, 1 genes
centered log2(fpkm+1)
2.0
1.5
1.0
0.5
0.0
−0.5
−1.0
CKF_1
CKF_2
CKF_3
CKM_1
CKM_2
CKM_3
ADF_1
ADF_2
ADF_3
ADM_1
ADM_2
ADM_3

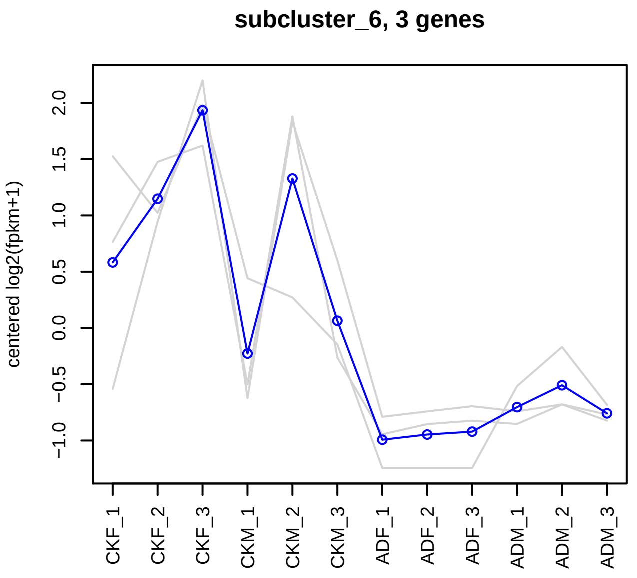
subcluster_6, 3 genes
centered log2(fpkm+1)
2.0
1.5
1.0
0.5
0.0
−0.5
−1.0
CKF_1
CKF_2
CKF_3
CKM_1
CKM_2
CKM_3
ADF_1
ADF_2
ADF_3
ADM_1
ADM_2
ADM_3

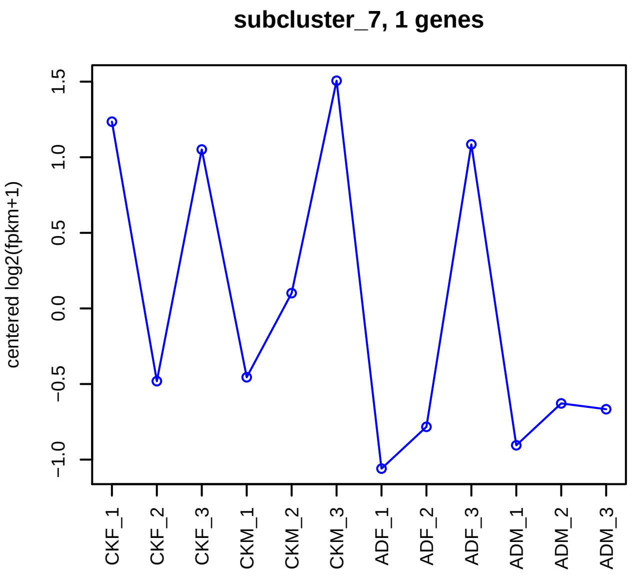
subcluster_7, 1 genes
centered log2(fpkm+1)
1.5
1.0
0.5
0.0
−0.5
−1.0
CKF_1
CKF_2
CKF_3
CKM_1
CKM_2
CKM_3
ADF_1
ADF_2
ADF_3
ADM_1
ADM_2
ADM_3

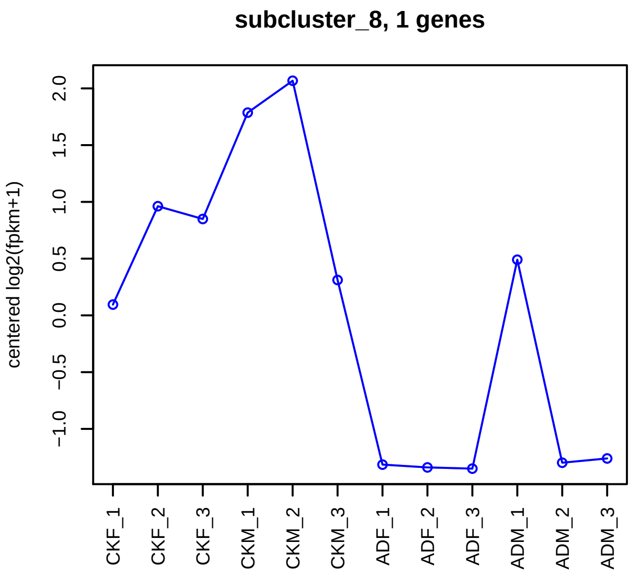
subcluster_8, 1 genes
centered log2(fpkm+1)
2.0
1.5
1.0
0.5
0.0
−0.5
−1.0
CKF_1
CKF_2
CKF_3
CKM_1
CKM_2
CKM_3
ADF_1
ADF_2
ADF_3
ADM_1
ADM_2
ADM_3

subcluster_9, 1 genes

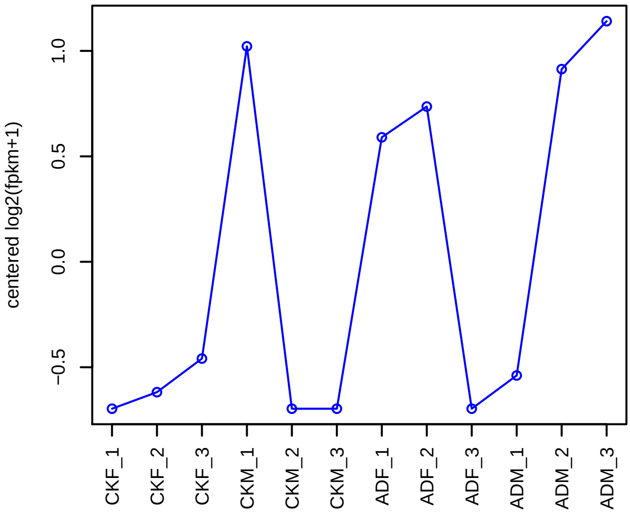

subcluster_10, 1 genes

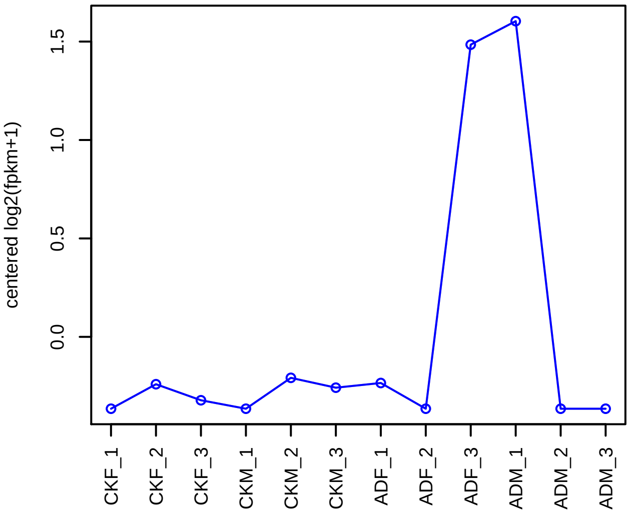

subcluster_10.xls, 1 genes

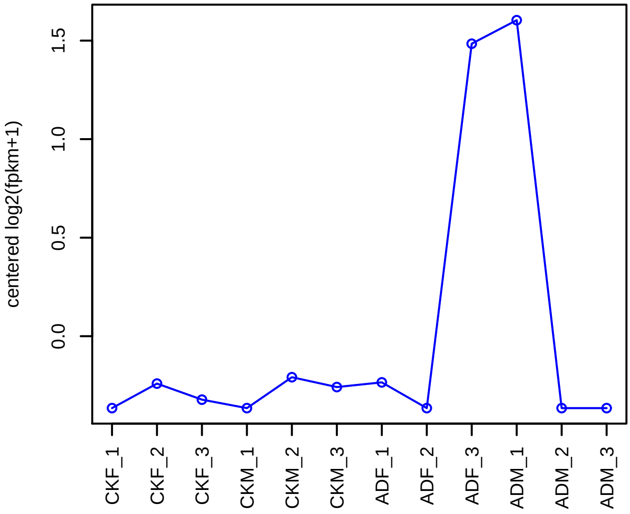

subcluster_1.xls, 1008 genes

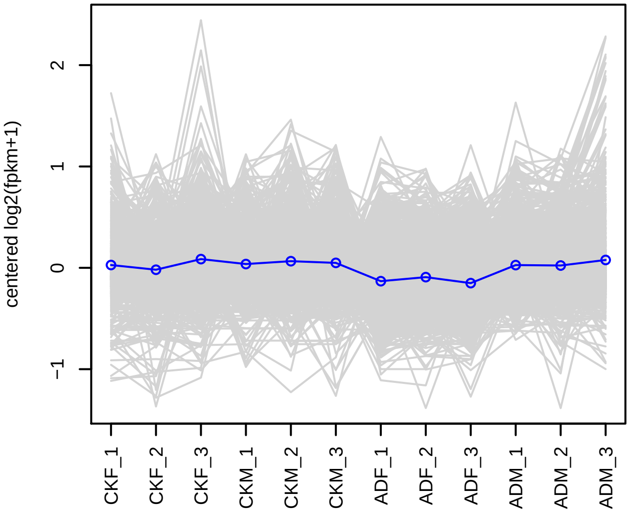

subcluster_2.xls, 3 genes

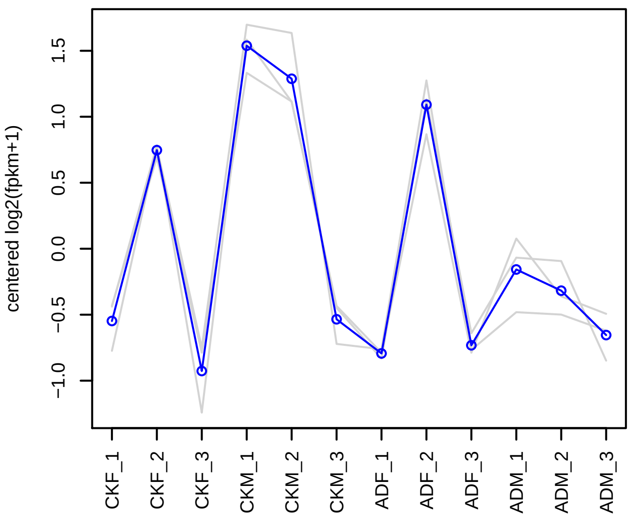

subcluster_3.xls, 71 genes

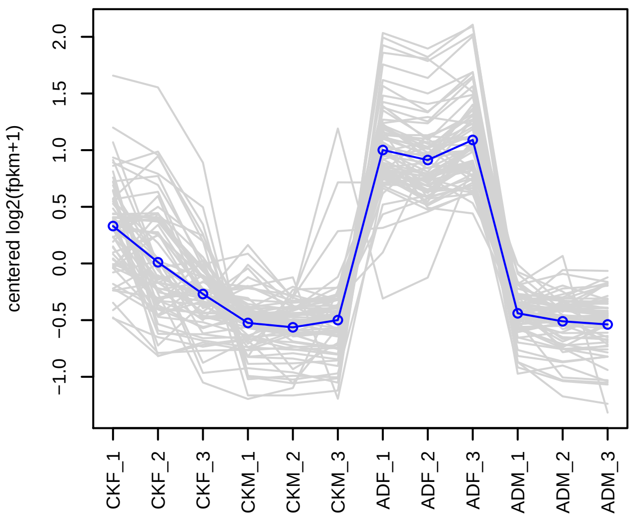

subcluster_4.xls, 7 genes

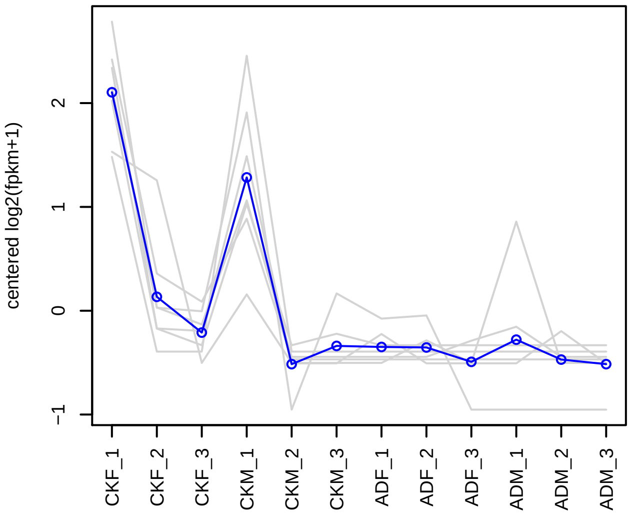

subcluster_5.xls, 1 genes

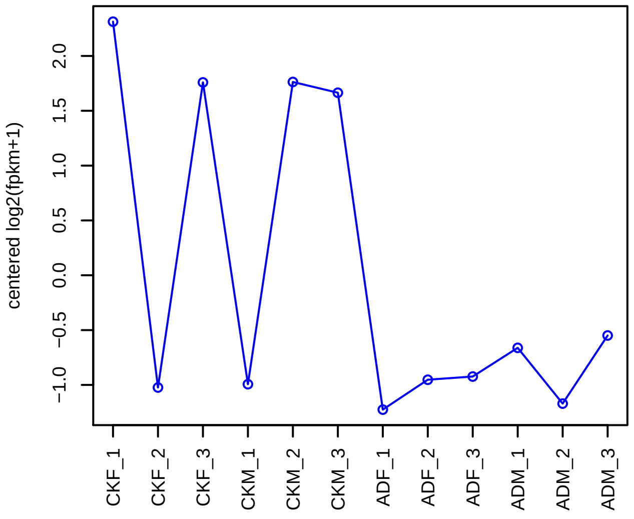

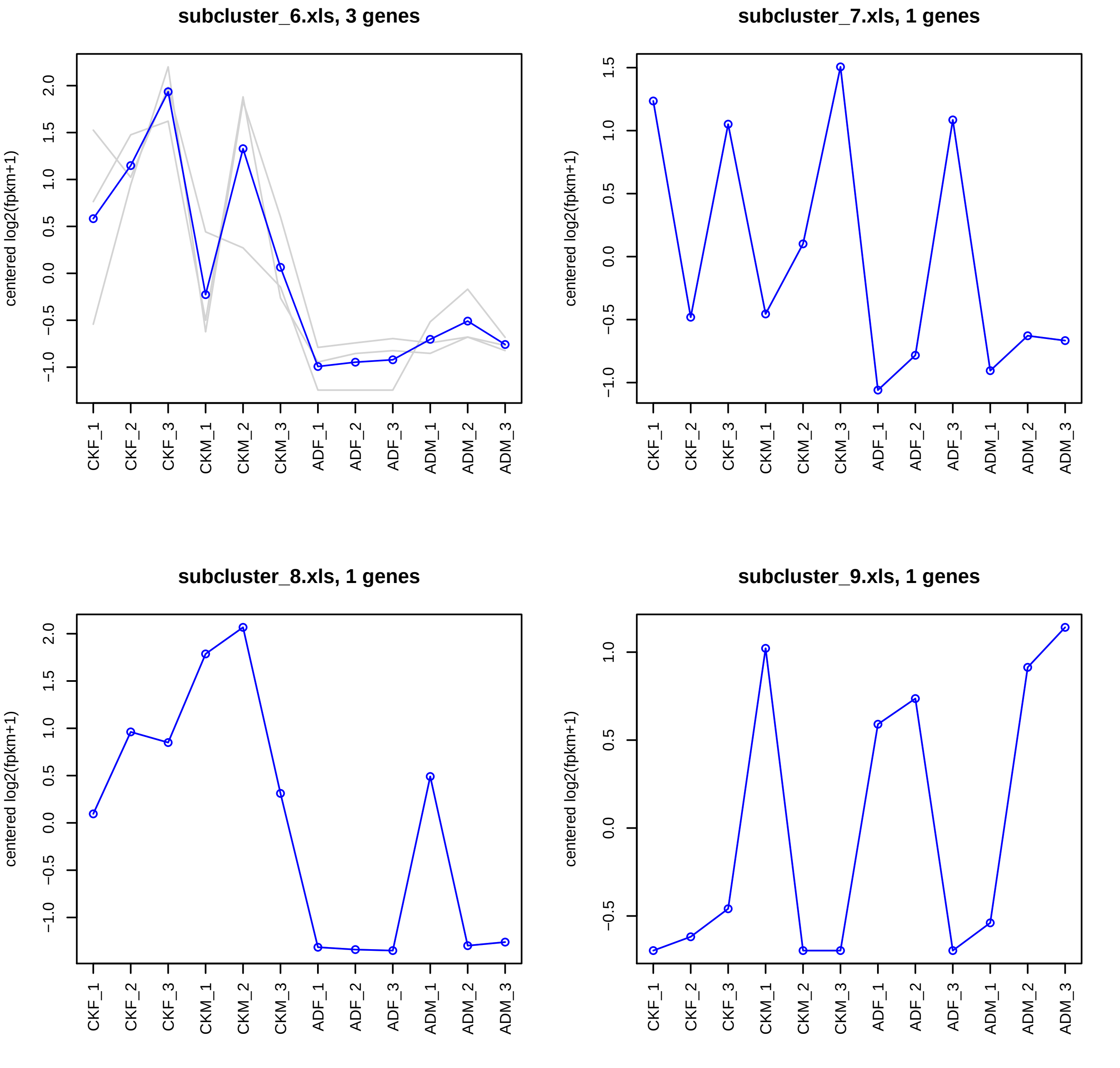

subcluster_6.xls, 3 genes
subcluster_7.xls, 1 genes
subcluster_8.xls, 1 genes
subcluster_9.xls, 1 genes
centered log2(fpkm+1)
CKF_1
CKF_2
CKF_3
CKM_1
CKM_2
CKM_3
ADF_1
ADF_2
ADF_3
ADM_1
ADM_2
ADM_3
