# Supplementary material for: Transcriptome sequencing of Coccinella septempunctata adults (Coleoptera: Coccinellidae) feeding on artificial diet and Aphis craccivora
Source: PLoS One. 2020 Aug 17;15(8):e0236249. doi: 10.1371/journal.pone.0236249 (PMC7430724; doi:10.1371/journal.pone.0236249)
Supplement: S6 File — (DOC) [file pone.0236249.s006.doc]

**Top eighty differentially expressed genes in libraries of *Coccinella septempunctata* artificial diet-fed (ADF/M)and *Aphis craccivora*-fed (CKF/M) treatments.**

| Unigene ID | Nr_ Description | Pfam | Fold change | | FDR |
| --- | --- | --- | --- | --- | --- |
| **ADF/CKF(up-regulated)** | |  |  |  | |
| | c92835_g1 | | --- | | hypothetical protein TcasGA2_TC005927 | PF03022.13|MRJP|Major royal jelly protein|m. 20219:116-358 | 9.25 | 9.96E-49 | |
| c91785_g2 | yellow-g precursor [Tribolium hypothetical protein TcasGA2_TC006226 | PF03022.13|MRJP|Major royal jelly protein|m. 41728:122-357 | 9.28 | 1.36E-48 | |
| | c96513_g1 | | --- | | _ | _ | 6.79 | 3.33E-33 | |
| c93731_g1 | hypothetical protein TcasGA2_TC014927 | _ | 8.90 | 2.46E-31 | |
| c98056_g1 | _ | _ | 5.62 | 9.37E-24 | |
| c84334_g1 | _ |  | 5.74 | 1.48E-20 | |
| | c99027_g1 | | --- | | microfilarial sheath protein SHP3 | _ | 4.84 | 4.17E-17 | |
| c28827_g1 | yellow-y precursor [Tribolium yellow-y | PF03022.13|MRJP|Major royal jelly protein|m. 27370:124-410 | 4.89 | 2.06E-13 | |
| c96662_g1 | _ |  | 6.61 | 1.13E-12 | |
| c75922_g2 | _ |  | 3.23 | 2.78E-12 | |
| | c90335_g1 | | --- | | hypothetical protein TcasGA2_TC007032 | PF00149.25|Metallophos|Calcineurin-like phosphoesterase|m.39311:179-442 | 3.14 | 1.13E-07 | |
| c95950_g1 | _ | _ | 4.91 | 1.16E-07 | |
| | c72893_g1 | | --- | | _ | _ | 5.69 | 1.39E-07 | |
| | c87662_g1 | | --- | | _ | _ | 4.85 | 1.52E-07 | |
| c99157_g1 | PREDICTED: nose resistant to fluoxetine protein 6-like | PF01757.19|Acyl_transf_3|Acyltransferase family|m.49689:281-644 | 4.61 | 2.70E-07 | |
| c89702_g1 | _ | _ | 5.47 | 2.88E-07 | |
| c63691_g1 | hypothetical protein VITISV_032906 | PF07727.11|RVT_2|Reverse transcriptase (RNA-dependent DNA polymerase)|m.43391:  791-1035;PF00665.23|rve|Integrase core domain|m.43391:446-558;  PF14223.3|Retrotran_gag_2|gag-polypeptide of LTR copia-type|m.43391:45-173;  PF13976.3|gag_pre-integrs|GAG-pre-integrase domain|m.43391:378-430;  PF07727.11|RVT_2|Reverse transcriptase (RNA-dependent DNA polymerase)|m.  43392:791-1035;  PF00665.23|rve|Integrase core domain|m.43392:  446-558;  PF14223.3|Retrotran_gag_2|gag-polypeptide of LTR copia-type|m.43392:45-173;  PF13976.3|gag_pre-integrs|GAG-pre-integrase domain|m.43392:378-430 | 3.64 | 4.98E-07 | |
| c92449_g1 | PREDICTED: uncharacteriz-  ed protein LOC101234439 | PF05970.11|PIF1|PIF1-like helicase|m.18483:  300-642;  PF13604.3|AAA_30|AAA domain|m.18483:  301-505 | 3.55 | 5.97E-07 | |
| c91793_g1 | _ | _ | 2.94 | 2.23E-06 | |
| c91828_g1 | PREDICTED: facilitated trehalose transporter Tret1-2 homolog | PF00083.21|Sugar_tr|Sugar (and other) transporter|m.41319:58-420;  PF07690.13|MFS_1|Major Facilitator Superfamily|m.41319:17-400 | 4.81 | 3.36E-06 | |
| c91942_g1 | uncharacterized protein LOC100903664 | PF14214.3|Helitron_like_N|Helitron helicase-like domain at N-terminus |m.42412:568-752;  PF04843.9|Herpes_teg_N|Herpesvirus tegument protein, N-terminal conserved region  |m.42412:64-217 | 3.36 | 3.40E-06 | |
| c83553_g1 | _ | _ | 5.24 | 5.77E-06 | |
| c85074_g1 | Lipase 3 precursor, putative | PF04083.13|Abhydro_lipase|Partial alpha/beta-hydrolase lipase region|m.  40016:32-91;  PF00561.17|Abhydrolase_1|alpha/beta hydrolase fold|m.40016:72-200 | 3.85 | 5.80E-06 | |
| c95818_g1 | _ | _ | 4.25 | 7.00E-06 | |
| c92486_g1 | _ | _ | 4.07 | 7.00E-06 | |
| c100740_g1 | hypothetical protein TcasGA2_TC007131 | _ | 2.57 | 7.72E-06 | |
| c99100_g1 | _ | _ | 2.78 | 1.22E-05 | |
| c124434_g1 | _ | _ | 5.25 | 1.87E-05 | |
| c92435_g2 | _ | _ | 3.96 | 1.87E-05 | |
| c86781_g1 | PREDICTED: pancreatic lipase-related protein 2-like | PF00151.16|Lipase|Lipase|m.45433:30-284 | 5.49 | 2.28E-05 | |
| **ADF/CKF(down-regulated)** | |  |  |  | |
| | c91042-g1 | | --- | | _ | _ | -8.57 | 5.37E-23 | |
| c87825_g1 | _ | PF14223.3|Retrotran_gag_2|gag-polypeptide of LTR copia-type|m.17932:158-285 | -6.79 | 4.71E-21 | |
| c89981_g1 | ORF1 [Aphid lethal paralysis virus] | PF00680.17|RdRP_1|RNA dependent RNA polymerase|m.38283:1580-1965;  PF00910.19|RNA_helicase|RNA helicase|m.  38283:567-679;  PF08762.7|CRPV_capsid|CRPV capsid protein like|m.38284:591-797;  PF00073.17|Rhv|picornavirus capsid protein|m.  38284:51-174;  PF00073.17|Rhv|picornavirus capsid protein|  m.38284:403-480;  PF11492.5|Dicistro_VP4|Cricket paralysis virus, VP4|m.38284:240-291 | -7.21 | 4.17E-18 | |
| c86394_g1 | _ | _ | -5.49 | 3.11E-11 | |
| c86222_g3 | _ | PF01395.19|PBP_GOBP|PBP/GOBP family|m.37204:33-124 | -3.38 | 3.65E-09 | |
| c90420_g1 | PREDICTED: uncharacteriz-  ed protein LOC659913 | _ | -5.98 | 3.73E-09 | |
| c79805_g2 | PREDICTED: protein spalten  -like | _ | -13.12 | 2.03E-08 | |
| c41266_g1 | _ | _ | -6.91 | 3.86E-08 | |
| c82424_g2 | hypothetical protein TcasGA2_TC009388 | PF12017.5|Tnp_P_element|Transposase protein|m.45862:24-174 | -5.01 | 1.26E-07 | |
| c100113_g1 | PREDICTED: toll-like receptor 2 | PF13855.3|LRR_8|Leucine rich repeat|m.24500:  85-145;  PF13855.3|LRR_8|Leucine rich repeat|m.  24500:293-349;  PF13855.3|LRR_8|Leucine rich repeat|m.24500:  368-427;  PF13855.3|LRR_8|Leucine rich repeat|m.24500:  469-509;  PF01582.17|TIR|TIR domain|m.24500:676-818;  PF13306.3|LRR_5|Leucine rich repeats (6 copies)  |m.24500:72-143;  PF13306.3|LRR_5|Leucine rich repeats (6 copies)|  m.24500:362-425;  PF13306.3|LRR_5|Leucine rich repeats (6 copies)|  m.24500:475-551;  PF13676.3|TIR_2|TIR domain|m.24500:677-754 | -3.1 | 1.39E-07 | |
| c97111_g3 | _ | _ | -4.04 | 1.45E-07 | |
| c95404_g1 | PREDICTED: esterase FE4 | PF00135.25|COesterase|Carboxylesterase family|m.486:38-560 | -4.17 | 5.66E-07 | |
| c87947_g1 | PREDICTED: uncharacteriz-  ed protein LOC656417 isoform X2 | _ | -3.15 | 6.28E-07 | |
| c95197_g5 | _ | _ | -7.16 | 6.28E-07 | |
| c89293_g1 | _ | _ | -2.93 | 9.53E-07 | |
| c85961_g2 | _ | _ | -2.53 | 1.16E-06 | |
| c86442_g1 | PREDICTED: uncharacteriz-  ed protein LOC657063 | PF03067.12|LPMO_10|Lytic polysaccharide mono-oxygenase, cellulose-degrading|m.45505:21-211 | -2.48 | 1.43E-06 | |
| c95171_g1 | _ | PF00050.18|Kazal_1|Kazal-type serine protease inhibitor domain|m.604:60-100 | -2.91 | 1.61E-06 | |
| c79963_g1 | pherophorin-dz1 protein | _ | -3.31 | 1.79E-06 | |
| c84392_g1 | _ | _ | -2.55 | 1.97E-06 | |
| c99532_g1 | Gag-Pol polyprotein | PF07727.11|RVT_2|Reverse transcriptase (RNA-dependent DNA polymerase)|m.  23411:823-1062;  PF14223.3|Retrotran_gag_2|gag-polypeptide of LTR copia-type|m.23411:51-186;  PF00665.23|rve|Integrase core domain|m.23411:  476-592;  PF13976.3|gag_pre-integrs|GAG-pre-integrase domain|m.23411:392-462;  PF00098.20|zf-CCHC|Zinc knuckle|m.23411:  230-244 | -3.63 | 2.23E-06 | |
| c34688_g1 | hypothetical protein | _ | -9.88 | 2.41E-06 | |
| c77938_g1 | _ | PF07464.8|ApoLp-III|Apolipophorin-III precursor (apoLp-III)|m.28019:55-183 | -10.15 | 2.81E-06 | |
| c90002_g1 | PREDICTED: farnesyl pyrophosphate synthase | PF00348.14|polyprenyl_synt|Polyprenyl synthetase|m.39103:72-327 | -3.74 | 3.09E-06 | |
| c95537_g1 | _ | _ | -3.27 | 3.39E-06 | |
| c90640_g2 | _ | _ | -4.4 | 4.73E-06 | |
| c95406_g1 | hypothetical protein D910_  06185 | PF13927.3|Ig_3|Immunoglobulin domain|m.2364:  153-227;  PF13927.3|Ig_3|Immunoglobulin domain|m.2364:  256-321;  PF07679.13|I-set|Immunoglobulin I-set domain|m.  2364:157-233;  PF07679.13|I-set|Immunoglobulin I-set domain|m.  2364:254-332;  PF00047.22|ig|Immunoglobulin domain|m.2364:  163-235;  PF00047.22|ig|Immunoglobulin domain|m.2364:  257-327;  PF13895.3|Ig_2|Immunoglobulin domain|m.2364  :166-236;  PF13895.3|Ig_2|Immunoglobulin domain|m.2364:  262-328 | -3.08 | 6.29E-06 | |
| c83715_g1 | hypothetical protein TcasGA2_TC001688 | _ | -4.18 | 6.75E-06 | |
| c63757_g1 | _ | _ | -3.88 | 6.77E-06 | |
| c86831_g1 | _ | _ | -3.83 | 6.77E-06 | |
| **ADM/CKM(up-regulated)** | |  |  |  | |
| c93955_g1 | hypothetical protein YQE_10095, partial | PF03062.16|MBOAT|MBOAT, membrane-bound O-acyltransferase family|m.5478:104-432 | 6.35 | 3.16E-18 | |
| c53169_g1 | cytochrome P450 CYP6BQ21 | PF00067.19|p450|Cytochrome P450|m.  40184:46-509 | 6.27 | 1.74E-11 | |
| c65983_g1 | hypothetical protein EAI_  17469 | _ | 8.76 | 4.58E-07 | |
| c57249_g1 | _ | _ | 11.56 | 1.02E-06 | |
| c95225_g1 | _ | _ | 5.7 | 1.04E-06 | |
| | c75846_g1 | | --- | | _ | _ | 6.62 | 6.57E-06 | |
| c78103_g1 | PREDICTED: glycine-rich protein | _ | 8.78 | 6.57E-06 | |
| c118425_g1 | _ | _ | 6.46 | 7.63E-06 | |
| c84550_g1 | PREDICTED: apolipoprotein D-like | PF00061.20|Lipocalin|Lipocalin / cytosolic fatty-acid binding protein family|m.39583:97-240;  PF08212.9|Lipocalin_2|Lipocalin-like domain|m.  39583:90-218 | 2.77 | 7.63E-06 | |
| c85264_g1 | _ | _ | 4.55 | 8.62E-06 | |
| **ADM/CKM(down-regulated)** | |  |  |  | |
| | c87825_g1 | | --- | | _ | PF14223.3|Retrotran_gag_2|gag-polypeptide of LTR copia-type|m.17932:158-285 | -6.11 | 2.48E-16 | |
| c86471_g2 | _ | _ | -5.88 | 1.04E-06 | |
| c75122_g1 | _ | _ | -6.5 | 2.08E-06 | |
| c82424_g2 | hypothetical protein TcasGA2_TC009388 | PF12017.5|Tnp_P_element|Transposase protein|m.45862:24-174 | -3.94 | 2.26E-06 | |
| c77938_g1 | _ | PF07464.8|ApoLp-III|Apolipophorin-III precursor (apoLp-III)|m.28019:55-183 | -8.46 | 4.12E-05 | |
| c96157_g2 | _ | PF12017.5|Tnp_P_element|Transposase protein|m.  33868:23-231;PF12596.5|Tnp_P_element_C|87kDa Transposase|m.33868:488-583 | -4.08 | 6.40E-05 | |
| c90563_g3 | _ | _ | -4.32 | 8.06E-05 | |
| c97725_g1 | _ | _ | -4.38 | 8.31E-05 | |
| c88602_g1 | PREDICTED: general odorant-binding protein 19d-like | PF01395.19|PBP_GOBP|PBP/GOBP family|m.  12124:20-135 | -7.56 | 1.10E-04 | |
| c63757_g1 | _ | _ | -2.47 | 1.15E-04 | |
