# Supplementary material for: Transcriptome sequencing of Coccinella septempunctata adults (Coleoptera: Coccinellidae) feeding on artificial diet and Aphis craccivora
Source: PLoS One. 2020 Aug 17;15(8):e0236249. doi: 10.1371/journal.pone.0236249 (PMC7430724; doi:10.1371/journal.pone.0236249)
Supplement: S13 File — (DOC) [file pone.0236249.s013.doc]

**Differentially expressed genes related to different biological characteristics in libraries of *Coccinella septempunctata* fed on artificial diet (ADF/M) compared to *Aphis craccivora* (CKF/M).**

| Pfam | Unigene_ ID | NR_ Description | ︱Log2FC︱﹥=1(♀) | ︱Log2FC︱﹥=1(♂) | FDR(♀) | FDR (♂) |
| --- | --- | --- | --- | --- | --- | --- |
| Haemolymph juvenile hormone binging protein | c90870_g2 | PREDICTED: protein takeout-like [Diaphorina citri] | -2.58 |  | 6.94E-03 |  |
|  | c80106_g1 | PREDICTED: protein takeout isoform X1 [Tribolium castaneum] | 3.12 |  | 4.80E-02 |  |
|  | c95505_g1 | conserved hypothetical protein [Culex quinquefasciatus] | -2.71 |  | 1.23E-02 |  |
|  | c90428_g1 | hypothetical protein YQE_06238, partial [Dendroctonus ponderosae] | 2.1 | 1.42 | 4.77E-02 | 7.59E-01 |
|  | c90925_g1 | PREDICTED: circadian clock-controlled protein [Tribolium castaneum] | -1.62 |  | 1.97E-02 |  |
|  | c93416_g1 | hypothetical protein TcasGA2_TC001181 [Tribolium castaneum] | -2.33 |  | 3.88E-03 |  |
|  | c91985_g1 | PREDICTED: protein takeout-like [Tribolium castaneum] | -2.56 |  | 1.35E-04 |  |
|  | c85733_g1 | AGAP011167-PA [Anopheles gambiae str. PEST] | 0.37 | 2.3 | 8.40E-01 | 3.19E-02 |
| [Ldl_recept_a] Low-density lipoprotein receptor domain class | c100390_g1 | vitellogenin receptor [Rhyparobia maderae] | 2.49 |  | 2.66E-03 |  |
| [NAD_binding_4]Male sterility protein | c91029_g1  c100017_g1 | AGAP011736-PA-like protein [Anopheles sinensis]  PREDICTED: putative fatty acyl-CoA reductase CG5065 [Tribolium castaneum] | -7.61  -1.92 |  | 2.11E-04  4.58E-02 |  |
| [Vitellogenin_N] Lipoprotein  amino terminal region | c98041_g1 |  | 1.75 |  | 3.80E-05 |  |
| Cytochrome P450 | c89051_g1 | cytochrome P450 345D2 [Tribolium castaneum] | 1.27 |  | 7.53E-03 |  |
|  | c93847_g2 | cytochrome P450 CYP4BN1 [Tribolium castaneum] | 2.87 |  | 3.64E-02 |  |
|  | c86966_g1 | cytochrome P450 307A1 [Tribolium castaneum] | 2.03 |  | 2.70E-02 |  |
|  | c53169_g1 | cytochrome P450 CYP6BQ21 [Dastarcus helophoroides] | 1.33 | 6.27 | 5.97E-01 | 1.74E-11 |
|  | c94356_g1 | cytochrome P450 315a1 [Leptinotarsa decemlineata] | 1.38 |  | 4.13E-02 |  |
|  | c88107_g1 | cytochrome P450 [Tribolium castaneum] | 2.72 | -1.51 | 3.24E-03 | 7.78E-01 |
|  | c100693_g1 | PREDICTED: cytochrome P450 9Z4 isoform X1 [Tribolium castaneum] | -2.35 |  | 3.09E-03 |  |
| [zf-C4] Zinc finger, C4 type  (two domains) | c100510_g1 | thr4 [Tenebrio molitor] | 2.06 | 1.05 | 2.72E-02 | 6.87E-01 |
|  | c100070_g1 | ecdysone-induced protein 78c [Haliotis diversicolor] | 1.57 |  | 1.35E-02 |  |
|  | c100898_g1 | PREDICTED: probable nuclear hormone receptor HR38 [Tribolium castaneum] | -2.69 |  | 2.97E-02 |  |
|  | c90602_g1 | PREDICTED: nuclear receptor subfamily 2 group E member 1 [Tribolium castaneum] | 3.52 | 1.64 | 3.21E-02 | 4.89E-01 |
| [PBF_GOBP]PBP/GOBP family | c86409_g1 | PREDICTED: B1 protein [Tribolium odorant binding protein (subfamily minus-C) C04 [Tribolium castaneum] | -2.03 |  | 3.71E-03 |  |
|  | c88602_g1 | PREDICTED: general odorant-binding protein 19d-like [Tribolium castaneum] | -2.35 | -7.56 | 7.22E-05 | 1.10E-04 |
|  | c83801_g1 | PREDICTED: general odorant-binding protein 19d-like [Tribolium castaneum] | -2.35 | -1.05 | 7.22E-05 | 2.14E-01 |
|  | c84691_g1 | PREDICTED: B1 protein [Tribolium odorant binding protein (subfamily minus-C) C04 [Tribolium castaneum] | -2.44 | -1.75 | 1.21E-03 | 6.53E-03 |
| [MRJP] Major royal jelly protein | c93776_g1 | yellow-c precursor [Tribolium hypothetical protein TcasGA2_TC016299 [Tribolium castaneum] | -2.38 |  | 6.30E-04 |  |
|  | c91785_g2 | yellow-g precursor [Tribolium hypothetical protein TcasGA2_TC006226 [Tribolium castaneum] | 9.28 |  | 1.36E-48 |  |
|  | c28827_g1 | yellow-y precursor [Tribolium yellow-y [Tribolium castaneum] | 4.89 |  | 2.06E-13 |  |
|  | c92835_g1 | hypothetical protein TcasGA2_TC005927 [Tribolium castaneum] | 9.25 |  | 9.96E-49 |  |
|  | c68563_g1 | PREDICTED: yellow-e3 isoform X1 [Tribolium castaneum] | -2.67 |  | 1.47E-04 |  |
| [OS-D] Insect pheromone-binding family,A10/OS-D | c72083_g1 | putative chemosensory binding protein [Nylanderia nr. pubens LZ-2010] |  | -4.61 |  | 2.07E-02 |
|  | c84975_g1 | PREDICTED: transcriptional adapter 2B-like [Megachile rotundata] |  | -2.59 |  | 4.97E-02 |
|  | c86374_g1 | serine/threonine kinase [Anopheles darlingi] | -2.37 |  | 3.04E-03 |  |
|  | c88732_g1 | chemosensory protein 12 precursor [Tribolium castaneum] | -3.62 |  | 2.87E-02 |  |
|  | c57475_g1 | protein serine/threonine kinase, putative [Aedes aegypti] | -4.33 |  | 4.43E-02 |  |
|  | c87581_g1 | putative chemosensory binding protein [Stomoxys calcitrans] | -1.59 |  | 3.36E-02 |  |
