# Supplementary material for: Transcriptome sequencing of Coccinella septempunctata adults (Coleoptera: Coccinellidae) feeding on artificial diet and Aphis craccivora
Source: PLoS One. 2020 Aug 17;15(8):e0236249. doi: 10.1371/journal.pone.0236249 (PMC7430724; doi:10.1371/journal.pone.0236249)
Supplement: S14 File — (DOC) [file pone.0236249.s014.doc]

**KEGG of DEG related to nutrients of artificial diets.**

| Nutrients | pathway | Pathway Id | DEGs genes with  pathway annotation | | All genes with pathway annotation | P-Value | | Corrected P-Value | |
| --- | --- | --- | --- | --- | --- | --- | --- | --- | --- |
|  |  |  | ADF vs. CKF | ADM vs. CKM |  | ADF vs. CKF | ADM vs. CKM | ADF vs. CKF | ADM vs. CKM |
| Amino acid | glycine, serine and threonine metabolism | Ko00260 | 19(1↑, 18↓) | 13(6↑, 7↓) | 191 | 0.213311 | 0.050133 | 0.798387 | 0.549371 |
|  | valine, leucine and isoleucine biosynthesis | Ko00290 | 1(0↑, 1↓) |  | 41 | 0.960575 |  | 1 |  |
|  | cysteine and methionine metabolism | Ko00270 | 6(2↑, 4↓) | 2(0↑,2↓) | 152 | 0.979383 | 0.989435 | 1 | 1 |
|  | lysine biosynthesis | ko00300 | 1(1↑, 0↓) | 1(↑, 0↓) | 33 | 0.926988 | 0.766392 | 1 | 1 |
|  | tryptophan metabolism | ko00380 | 10(2↑, 8↓) | 6(3↑, 3↓) | 145 | 0.719018 | 0.608781 | 1 | 1 |
|  | glutathione metabolism | ko00480 | 16( 0↑, 16↓) | 5(2↑, 3↓) | 120 | 0.044218 | 0.603789 | 0.309529 | 1 |
|  | lysine degradation | ko00310 | 11(1↑, 10↓) | 9(5↑, 4↓) | 142 | 0.585102 | 0.182723 | 1 | 0.862831 |
|  | biosynthesis of amino acids | ko01230 | 16(2↑, 14↓) | 8(6↑, 2↓) | 436 | 0.999824 | 0.998398 | 1 | 1 |
| fat | adipocytokine signaling pathway | ko04920 | 6(0↑, 6↓) | 5(2↑, 3↓) | 59 | 0.34988 | 0.129421 | 1 | 0.791577 |
|  | pyruvate metabolism | ko00620 | 11(0↑, 11↓) | 1( 0↑, 1↓) | 259 | 0.991425 | 0.999986 | 1 | 1 |
|  | fatty acid biosynthesis | ko00061 | 2(0↑, 2↓) | 4(3↑, 1↓) | 58 | 0.942801 | 0.260728 | 1 | 0.992515 |
|  | glycerolipid metabolism | ko00561 | 14(3↑, 11↓) | 5(2↑, 3↓) | 90 | 0.021203 | 0.366166 | 0.172486 | 1 |
|  | fat digestion and absorption | ko04975 | 7(5↑, 2↓) | 3(2↑, 1↓) | 30 | 0.017422 | 0.158559 | 0.163875 | 0.851308 |
|  | fatty acid metabolism | ko01212 | 10(2↑, 8↓) | 6(4↑, 2↓) | 162 | 0.826973 | 0.709271 | 1 | 1 |
|  | biosynthesis of unsaturated fatty acids | ko01040 | 5(2↑, 3↓) | 1(0↑, 1↓) | 44 | 0.296035 | 0.85411 | 0.918624 | 1 |
| Starch and sugar | starch and sucrose metabolism | ko00500 | 15(3↑, 12↓) | 20(12↑, 8↓) | 189 | 0.549863 | 0.000526 | 1 | 0.013832 |
|  | carbohydrate digestion and absorption | ko04973 | 9(0↑, 9↓) | 5(4↑, 1↓) | 47 | 0.021075 | 0.065607 | 0.17248573 | 0.567455 |
| vitamin | ascorbate and aldarate metabolism | ko00053 | 10(3↑, 7↓) | 13(5↑, 8↓) | 92 | 0.222806 | 0.000445 | 0.798387 | 0.013009 |
|  | vitamin digestion and absorption | ko04977 | 3(1↑, 2↓) | 1(0↑, 1↓) | 16 | 0.162335 | 0.516573 | 0.746926 | 1 |
|  | folate biosynthesis | ko00790 | 4(1↑, 3↓) | 1(0↑, 1↓) | 39 | 0.395462 | 0.819294 | 1 | 1 |
|  | retinol metabolism | ko00830 | 9(3↑, 6↓) | 12(4↑, 8↓) | 75 | 0.168315 | 0.000268 | 0.756161 | 0.011729 |
|  | one carbon pool by folate | ko00670 | 3(1↑, 2↓) | 4(4↑, 0↓) | 65 | 0.887963 | 0.326909 | 1 | 1 |
|  | porphyrin and chlorophyll metabolism | ko00860 | 11(3↑, 8↓) | 12(4↑, 8↓) | 173 | 0.809075 | 0.088992 | 1 | 0.650137 |
